# HIV Infection in Fishing Communities of Lake Victoria Basin of Uganda – A Cross-Sectional Sero-Behavioral Survey

**DOI:** 10.1371/journal.pone.0070770

**Published:** 2013-08-05

**Authors:** Alex Opio, Michael Muyonga, Noordin Mulumba

**Affiliations:** Department of National Disease Control, Ministry of Health, Kampala, Uganda; University of Ottawa, Canada

## Abstract

**Background:**

Uganda's first AIDS case was reported in a fishing village. Thereafter, due to varying risk factors, the epidemic spread heterogeneously to all regions, with some populations more affected. Given the recent rising trends in HIV infection in Uganda, it is crucial to know the risk factors in different populations. The aim of this study was to determine the prevalence and risk factors of HIV infection among fishing communities.

**Methodology:**

A cross-sectional survey of 46 fishing communities was conducted in 2010. Following written consent, 911 randomly selected respondents age 15–59 years were interviewed and gave blood for HIV testing. HIV testing was conducted in the field and central laboratory according to national algorithm. Survey protocol was approved by the Science and Ethics Committee of Uganda Virus Research Institute, and cleared by Uganda National Council for Science and Technology. Data was captured by EPIINFO and statistical analysis done in SPSS.

**Findings:**

Overall HIV prevalence was 22%; there was no difference by sex (*x* ^2^ test, p>0.05). Association with HIV infection was determined by *x* ^2^ test, p<0.5. Never married respondents had lower HIV prevalence (6.2%) than the ever married (24.1%). HIV prevalence was lower in younger respondents, age 15–24 years (10.8%) than in age group 25 years and above (26.1%). Muslims had lower HIV prevalence (14.4%) than Christians (25.2%). HIV prevalence was higher among respondents reporting 3 or more lifetime sexual partners (25.3%) than in those reporting less numbers (10.8%). HIV prevalence was higher among uncircumcised men (27%) than in circumcised men (11%). Multivariate analysis identified 4 risk factors for HIV infection; age, religion, ever condom use and number of lifetime sexual partners.

**Conclusions:**

HIV prevalence in the surveyed communities was three times higher than of general population. This underscores the need for tailor made HIV combination prevention interventions targeting fishing communities.

## Introduction

In Uganda, the first case of acquired immune deficiency syndrome (AIDS – Slim disease) was reported in 1982 in Kansensero, a fishing village located in the Southwestern part of the country [1]. Since then, the HIV/AIDS epidemic has spread to all parts of the country, with some geographic areas and population sectors more affected than others [2]. Variability in the underlying factors to HIV infection partly explains why some population sectors are at higher risk of human immune virus (HIV) infection than others. For instance, it has been observed that any kind of profession or work which involves migration and being away from home for long makes people more vulnerable to HIV transmission [3], [4]. Fishers fall among these categories of people. Given the recent trends in HIV infection in Uganda [5], it is crucial to establish the extent to which population sectors such as fishing communities are contributing to the epidemic. The 2011 Uganda AIDS Indicators Survey shows that the adult general population HIV prevalence rose from 6.4 to 7.3 percent between 2005 and 2011. The survey further shows that different parts of the country and population sectors are affected heterogeneously with the central and mid-northern regions of the country having the highest HIV prevalence.

Estimation of HIV prevalence among fishing communities is very important because of the tangible contributions that are made by the fish industry to national economic development. Fishing contributes to food security, as well as income that boost the domestic product. At the level of local fishing communities, fishing has a pivotal role in the survival of people. A regional study done in the Lake Victoria Basin of Uganda, Kenya and Tanzania showed that the main source of household income at the beaches comes from fisheries. Fisheries on the average were found to be the main source of household income for 87% of respondents [6]. Therefore, there is need to sustain the capacity for fishing by ensuring that fishing communities are healthy and HIV free. The HIV/AIDS epidemic has been recognized by fishing communities to be a hindrance to fishing industry [7], [8]. Akumu and colleagues reported that high incidences of HIV-related diseases crippled fishing activities around Lake Albert. This position was further underscored by what some of the surveyed fishers personally expressed. They said that sometimes they would not have enough energy to carry out comprehensive fishing and instead they would spend a lot of time out of fishing activities, one to several months and that fishing hours were significantly reduced.

In order to guide the planning of HIV/AIDS services that target fishing communities in the Lake Victoria Basin, the Lake Victoria Basin Commission (LVBC) took an initiative in 2010 to support generation of data on HIV prevalence and HIV-related behavioral risks in the fishing communities located in the area. Specifically, LVBC commissioned baseline studies in Uganda, Kenya and Tanzania with four major dimensions; namely, to determine a) HIV sero-prevalence, (b) behavioral risks among the populations of focus, (c) service availability and utilization, and (d) coordination, institutional policies and structures. At the time, comprehensive HIV information on fishing communities was found lacking. Furthermore, most of the HIV interventions in the region, including in Uganda were found to be targeting the general population as a whole leaving the fishing communities inadequately targeted. Through this survey, LVBC sought to bridge the aforementioned information gap. Subsequently, the information generated would inform the establishment of a framework for improving the effectiveness of the HIV/AIDS responses targeting mobile populations, including migrant workers in fisheries and agricultural plantations.

The main aim of this study was to determine the prevalence of HIV infection and HIV-related risks among the fishing communities of the Lake Victoria Basin of Uganda. The study has shed light on HIV status in fishing communities in LVB. In this paper, we present information that can be used to facilitate the planning of evidence-based framework for HIV prevention and control targeting fishing communities.

## Methodology

### Survey design

This was a cross-sectional survey. In each of the fishing community, household and individual interviews were carried out using structured questionnaires. Key informant interviews and focus group discussions were also conducted using topic guides. However, in this paper, we only report results of the household and individual interviews. The survey was conducted in August 2010.

### Setting

This sub-national survey was carried out in the Lake Victoria Basin of Uganda. The Lake Victoria Basin of Uganda has over 400 fish landing sites that are inhabited by fishing communities. A fishing community was defined as a social and economic group of persons living together in a locality and who derive their livelihood directly or indirectly from fishing activities. Members of the communities consisted of fishers (boat crew), boat owners, fish processors, boat makers, local fishing gear makers or repairers, fishing equipment dealers and managers, as well as fish mongers or traders.

### Study population

The inclusion criteria for the survey were women and men age 15–59 years, residence in any of the of 46 sampled fishing communities, direct or indirect involvement in fishing work or fish industry supporting work and consent for the survey.

### Sample size estimation

The required sample size was determined using the sample estimation formula by Kish Leslie [9]. Based on this formula, a minimum sample size of 920 was estimated to be adequate. In the sample size calculation, we assumed an HIV prevalence of 8.5 percent, permissible error of 2.5 percent, confidence intervals of 95 percent, design effect of 1.3 and a response rate of 70 percent.

### Sampling

A multi-stage sampling procedure was used to get the required sample. The Primary Sampling Unit (PSU) was a fish landing site and the Secondary Sampling Unit (SSU) was an individual residing in the landing site. A total of 46 landing sites were selected and in each, 20 members were selected, yielding an estimated sample size of 920 respondents. The 46 fish landing sites were selected from a total of 431 fish landing sites that existed then in the Lake Victoria Basin using a systematic sampling method. This approach ensured that a district which had more landing sites had a higher representation of landing sites (probability proportional to size).

Before the individual respondents were selected, the sampling frames of the 46 landing sites were updated to ensure that all residents and those engaged in earning their livelihood from fishing related work; and who were aged 15 years and above were registered. For the selection of the 20 individuals from each fish landing site, the following procedures were used; 1^st^, all the landing site members in each of the 46 selected landing sites were listed, 2^nd^, all the household heads located in the enumeration area where each of the selected landing site is located were listed, 3^rd^, the heads of the households were requested to list all the household members in the age bracket of 15–59 years. Arising from above, a final list of eligible respondents was obtained. From the final list, 20 respondents were selected using simple random sampling method.

#### Survey procedures

At the household level, heads of households were interviewed using household level survey questionnaire to determine eligible household members. Next, eligible individual household members were interviewed using a structured questionnaire. The questionnaire was adapted from that of the AIDS Indicator Survey (AIS). The AIS has been done in all the countries of East Africa and a number of other countries in sub-Saharan Africa. The questionnaire adaptation took into consideration the uniqueness of fishing communities. The questionnaires were translated into two main local languages spoken around Lake Victoria Basin and back translated into English. To ensure quality in all survey procedures, a number of quality control measures were taken. These measures included staff training, supervision of survey personnel and quality control of blood testing.

#### Data measures

The survey generated quantitative information on background characteristics (age, sex, ethnic grouping, level of education, marital status, for the married whether staying together with spouse, occupation, main source of income, length of stay in study area, mobility in the last 12 months), reproductive health, sexual activity, HIV-related behaviors, HIV-related knowledge, HIV-related attitudes, HIV/AIDS care services (range, breadth, availability, utilization) – HIV counseling and testing (HCT) services, condom availability, prevention of mother to child transmission (PMTCT), antiretroviral treatment (ART), opportunistic treatment (OI) treatment and health seeking behaviors for sexually transmitted infections.

#### Laboratory measures

After the questionnaires interviews, blood draw and field based laboratory testing of samples for HIV was conducted. Field based HIV testing was done to enable respondents know their results of HIV sero-status. Blood draw was done using capillary tubes. The technician collected 200 microlitre of blood from a fingerpick, using a capillary tube, for the tests that were to be performed in the field and 250 microliter on a filter paper card (dry blood spot – DBS) for central level testing. Blood tubes and specimen collection filter paper cards were labeled with the participant's bar-coded identification number (IDNO). Serum samples were tested by a two HIV EIA parallel testing algorithm (Vironostika HIV + Genetic Systems rLAV) following the manufacturer's recommendations and in accordance with national guidelines. Specimens with unambiguously positive or negative results on both assays were reported without further testing, while all others were re-tested by Western blot.

For quality control (QC) of blood testing, 5 percent of HIV positive and 10 percent of HIV negative specimens were re-tested at the HIV Reference Laboratory at the Uganda Virus Research Institute, Entebbe using the same testing algorithm. Specimens with discordant results were resolved by repeating the testing algorithm. The results of this quality control showed concordance with the testing done at the main central laboratory.

### Data management and analysis

Completed questionnaires were received at the central level (Survey Coordination Unit), registered and checked against the shipping inventory to verify that what was sent from the field had been received. Two data entry clerks entered the data using EPIINF0 following double data entry strategy. A statistician oversaw data entry and conducted data management and analysis. Data cleaning included the checking of ranges, structures and internal consistency.

We carried out bivariate and multivariate analysis using SPSS Version 16. Our main variable was HIV infection. In order to screen for variables to include in the multivariate model, variables had to undergo a first test to establish whether a specific variable had statistically significant association with HIV status using Chi-square (χ^2^) statistical test of significance. Only variables that were significantly associated with HIV infection at bivariate analysis were considered in the multivariate modeling. The predictor variables considered were age, religion, ever use of condoms and number of lifetime sexual partners. Backward elimination method was used to construct the final multivariate model. The final multivariate model shows all the predictor variables that are significantly associated with HIV infection at a level of p = <0.05.

### Ethics statement

The survey protocol was submitted to and approved by the Uganda Virus Research Institute Scientific and Ethical Committee (UVRI SEC), and cleared by the Uganda National Council for Science and Technology. Written informed consent was obtained from all participants for both individual interviews and blood draw and testing. Confidentiality was observed during filed interviews and laboratory testing. No personal identifiers were collected.

Prevention messages were provided by interviewers to all survey participants during the survey. All participants received HIV/STI education through person-to-person oral communication and/or in form of written HIV/STI education materials. Participants who consented to sample collection and HIV counseling and testing at the field level received free HIV counseling and testing.

### Findings

#### Background characteristics

Table 1 shows the characteristics of fishing community members. Of the 920 men and women selected for the survey, 911 (99 percent) were interviewed. Nine respondents (1.0 percent) were not interviewed because 6 (0.7 percent) refused interviews and 3 (0.3 percent) had moved away from the area for extended period during the survey. Of the 911 respondents interviewed, 893 (98 percent) agreed to the blood draw. Overall, the response rate was very high for both the interview and blood draw. About two thirds of the people interviewed, (559, and 61 percent) were men and one third (352, and 39 percent) were women. The majority of the respondents (88 percent) were in the age groups 20–44 years; only 6.4 percent of the respondents were in the age group 15–19 years.

**Table 1:** Characteristics of fishing community members age 15–59 years (N = 911).

Percent distribution of women and men by selected background characteristics			
	Female (N = 352)	Male (N = 559)	All (N = 911)
**Background characteristic**			
**Age**			
15–19	8.5	5.0	6.4
20–24	16.5	15.9	16.1
25–29	16.2	19.7	18.3
30–34	19.9	20.8	20.4
35–39	14.2	18.8	17.0
40–44	10.8	9.1	9.8
45–49	7.7	4.1	5.5
50–54	3.7	4.7	4.3
55–59	2.6	2.0	2.2
**Marital status**			
Never married	6.0	11.3	9.2
Married	51.4	61.4	57.5
Living together	18.2	14.0	15.6
Divorced/Separated	17.9	10.7	13.5
Widowed	6.5	2.7	4.2
**Education**			
Nursery	0.9	0.5	0.7
Primary	62.8	64.2	63.7
Post Primary/Vocational	3.7	4.3	4.1
Secondary/‘A’ Level	17.6	22.7	20.7
College	0	0.7	0.4
University	0.3	0.4	0.3
Not Stated	14.8	7.2	10.1
**Religion**			
Roman Catholic	37.2	38.3	37.9
Protestant/Other Christian	37.2	35.6	36.3
Muslim	16.5	21.5	19.5
No Religion	0.3	0.2	0.2
Other	8.5	4.5	6.0
**Distance to former place of residence**			
Born in that area	9.1	14.3	12.3
Less than 10 KM	6.0	6.3	6.1
10–29 KM	9.7	8.6	9.0
30–49 KM	14.5	9.8	11.6
50–99 KM	25.9	23.8	24.6
100 KM and above	34.1	36.5	35.6
Not Stated	0.9	0.7	0.8
**Mobility during last 12 months**			
Ever slept away	58.2	70.3	65.6
Never slept away	41.8	29.7	34.4
**Duration of Staying Away from Home During Last 12 Months**			
Ever been away for a month	13.9	23.6	19.9
Never been away for a month	86.1	76.4	80.1
**Total**	**100**	**100**	**100**

Slightly under two thirds (58 percent) of the respondents reported that they were married; while 16 percent said they were living together as man and wife, and only a very small proportion of respondents (4.2 percent) were widowed. Women were more likely to be widowed (6.5 percent) than men (2.7 percent). The majority of respondents (64 percent) stopped at primary school level and about a fifth (21 percent) had stopped at secondary school level, less than 1 percent of the respondents reached college or university.

The majority of the respondents were Christians (Roman Catholics 38 percent and Protestants 36 percent); one fifth (20 percent) of the respondents were Muslims. There was no apparent difference in the distribution of women and men by religion. The respondents were engaged in a variety of fishing related or fishing support work, namely, fish monger/factory agent (23.6 percent), food vendor (16.4 percent), barrier/worker (12.2 percent), boat owner/manager (8 percent), boat crew (6.6 percent), artisanal processor (2.4 percent), transporter (2.1 percent) and others (27.3 percent).

The majority of the respondents (88 percent) came from elsewhere other than their current places of residence; only 12 percent of the respondents were born in the current place of residence. The proportion of men and women who ever slept away from home during the last 12 months was 66 percent among all the respondents. In the last 12 months, men were more mobile (70 percent) than women (58 percent). Of all the respondents, 20 percent have ever stayed away from home for more than a month.

### HIV prevalence

Table 2 shows the estimated prevalence of HIV infection, as well as the prevalence of HIV-related knowledge, behavior and care variables among women and men age 15–49 years. The estimated HIV prevalence in both women and men age 15–49 years was 22 percent; HIV prevalence was higher among women (25.1%) than among men (20.5%).

**Table 2:** Estimated prevalence of HIV-related knowledge, behavior, care and biomarker variables among women and men age 15–49 years.

Variable	Women			Men		
	Number (Sample size) unweighted	Estimated Proportion (p)	95% Confidence Interval	Number (Sample size) unweighted	Estimated Proportion (p)	95% Confidence Interval
**Biomarker**						
HIV prevalence	346	0.251	0.193–0.310	547	0.205	0.157–0.252
**HIV-related Knowledge**						
Know that people can prevent getting the AIDS virus by using a condom every time they have sex	352	0.863	0.830–0.896	559	0.919	0.892–0.946
Know that people can prevent getting the AIDS virus by having one un infected partner who has no sexual intercourse with other partners	352	0.966	0.949–0.983	559	0.951	0.930–0.973
Able to cite that; people can prevent getting the AIDS virus by using condoms and limiting sex to one uninfected partner	352	0.841	0.807–0.875	559	0.878	0.844–0.912
Know that people can prevent getting the AIDS virus by abstaining from sexual intercourse	352	0.912	0.885–0.943	559	0.891	0.871–0.939
Comprehensive knowledge of HIV transmission	352	0.315	0.259–0.372	559	0.399	0.350–0.447
Know that a healthy-lookingperson can have the AIDS virus	352	0.903	0.865–0.942	559	0.911	0.881–0.940
Know that AIDS virus cannot be transmitted by mosquito bites	352	0.477	0.428–0.526	559	0.540	0.488–0.593
Know that AIDS virus cannot be transmitted by supernatural means	352	0.830	0.788–0.871	559	0.880	0.843–0.918
Know that AIDS virus cannot be transmitted by sharing utensils with someone who has AIDS	352	0.636	0.592–0.680	559	0.717	0.669–0.765
Rejects the two most common local misconceptions and say that a healthy-looking person can have the AIDS virus	352	0.355	0.298–0.413	559	0.428	0.374–0.481
Know that HIV can be transmitted by breastfeeding	352	0.835	0.787–0.883	559	0.732	0.688–0.775
Know that risk of MTCT can be reduced by mother taking special drugs during pregnancy	352	0.733	0.675–0.791	559	0.692	0.648–0.737
Know that HIV can be transmitted by breastfeeding and risk of MTCT can be reduced by mother taking special drugs during pregnancy	352	0.606	0.564–0.648	559	0.565	0.518–0.613
**HIV-related attitude**						
Willing to care for a family member with HIV at home	352	0.954	0.927–0.982	559	0.968	0.953–0.983
Would buy fresh vegetables or fish from a vendor who has HIV	352	0.869	0.834–0.903	559	0.848	0.813–0.883
Believe HIV positive female teacher should be allowed to keep teaching	352	0.755	0.705–0.805	559	0.778	0.743–0.813
Would not want HIV+ status of a family member to remain a secret	352	0.561	0.513–0.609	559	0.631	0.590–0.672
Accepting attitudes toward those living with HIV	352	0.399	0.353–0.445	559	0.452	0.410–0.493
**HIV-related behavior**						
Primary Abstinence: Respondents aged 15–24	88	0.068	−0.022–0.158	295	0.077	0.016–0.137
Secondary Abstinence: Sexually experienced respondents aged 15–24 years, that did not have sex in the past 12 months	82	0.024	−0.006–0.055	190	0.065	0.024–0.105
Had sex with more than one partner in the previous 12 months (15–49 years)	291	0.113	0.073–0.154	480	0.456	0.408–0.504
Higher risk sex in the last year (15–49 years)	291	0.241	0.182–0.299	303	0.425	0.372–0.478
Used condom at last higher risk sex (15–49 years)	291	0.471	0.368–0.575	204	0.593	0.510–0.676
Consistently used condom at higher risk sex in last 12 months	71	0.408	0.289–0.528	210	0.467	0.383–0.550
Had first sex by age 18 years	342	0.898	0.865–0.931	538	0.747	0.713–0.781
**HIV care**						
Tested for HIV in the last 12 months and received their test results the last time they were tested	352	0.364	0.303–0.425	559	0.308	0.246–0.369
Self-reporting an STI and/or symptoms of an STI in the last 12 months	346	0.237	0.185–0.289	550	0.180	0.149–0.211

#### HIV-related knowledge

As shown in table 2, HIV-related knowledge was high among respondents, 86 percent of women and 92 percent of men knew that people can prevent getting the AIDS virus by using a condom every time they have sex. Eighty six percent of women and 95 percent of men said that people can prevent getting the AIDS virus by having one uninfected partner who has no sexual intercourse with other partners. Eighty four percent of women and eighty eight percent of men were able to cite that that people can prevent getting the AIDS virus by using condoms and limiting sex to one uninfected partner. Almost all respondents were knowledgeable about the role of abstinence in HIV prevention; ninety one percent of women and eighty nine percent of men knew that people can prevent getting the AIDS virus by abstaining from sexual intercourse. Compared to knowledge of single methods for HIV prevention, the survey showed that comprehensive knowledge of HIV transmission was low among the respondents; it was 32 percent in women and 40 percent in men. Comprehensive knowledge of HIV is a composite variable that was defined as “knowing that consistent use of condom during sexual intercourse and having just one uninfected faithful partner can reduce the chance of getting the AIDS virus, knowing that a healthy-looking person can have the AIDS virus, and rejecting the two most common local misconceptions about transmission or prevention of the AIDS virus”.

Some members of the fishing communities still have misconceptions about HIV transmission. The proportion of respondents who knew that a healthy-looking person can have the AIDS virus was 90% in women and 91% in men. The proportion who knew that the AIDS virus cannot be transmitted by mosquito bites was slightly higher in men (54%) than in women (48%). Eighty three percent of women and 88% of men knew that the AIDS virus cannot be transmitted by supernatural means. Furthermore, 64% of women and 72% of women knew that the AIDS virus cannot be transmitted by sharing utensils with someone who has AIDS. Thirty six percent of women and 43% of men rejected the two most common local misconceptions and said that a healthy-looking person can have the AIDS virus.

Knowledge of PMTCT was moderate; 84% of women and 73% of men knew that HIV can be transmitted by breastfeeding. On the other hand, 73% of women and 69% of men knew that the risk of MTCT can be reduced by mother taking special drugs during pregnancy. The proportion of women and men who knew that HIV can be transmitted by breastfeeding and the risk of MTCT can be reduced by mother taking special drugs during pregnancy was 61% in women and 57% in men.

#### HIV-related attitudes

We assessed whether stigma-related attitudes existed among the respondents by asking four questions (table 2). The findings show that accepting attitudes toward those living with HIV was moderate, 40% of women and 45% of men had accepting attitudes. Specifically, 95% of women and 96% of men were willing to care for a family member with HIV at home, while 87% of women and 85% of men said they would buy fresh vegetables or fish from a vendor who has HIV. Fifty six percent of women and 63% of men believed that a HIV positive female teacher should be allowed to keep teaching. Whether HIV status should be revealed, 56% of women and 63% of men said they would not want HIV positive status of a family member to remain a secret.

#### HIV-related behaviors

HIV-related behaviors were also measured (table 2). At the analysis stage, youth were considered separately for some variables. Some small proportions of youth age 15–24 reported to be practicing primary abstinence; 7% of girls and 8% of boys said that they were practicing it. In the case of secondary abstinence; meaning, the proportion of sexually experienced respondents age 15–24 years that did not have sex in the past 12 months, it was lower in women (3%) than in men (7%). In regard to sexual debut, 90% of women and 75% of men said that they had first sex by age 18 years. Multiple sexual partnership was four times higher in men than in women; the proportion of the 15–49 year old women and men who had sex with more than one partner in the previous 12 months was 46% in men and 11% in women. Similarly, higher risk sex in the last year among the 15–49 year old was higher in men (43%) than in women (24%). Condom use was reported, among those age 15–49 years, 47% of women and 59% of men said they used condom at last higher risk sex. However, only 41% of women and 47% of men reported that they consistently used condom at higher risk sex in last 12 months.

#### HIV care

We also measured HIV care related variables; namely health seeking behaviours among those with self reported STI, and HIV counseling and testing. The findings (table 2), show that 36% of women and 31% of men reported that they were tested for HIV and received their test results the last time they were tested. Twenty four percent of women and 18% of men self-reported an episode of STI and/or symptoms of an STI in the last 12 months preceding the survey.

#### Bivariate analysis

Table 3 shows the estimated prevalence of HIV categorized by HIV-related variables among fishing community members. HIV prevalence varies significantly with age, religion, marital status and circumcision status of men. HIV infection was significantly higher in the age group 25 and above (26.1%) compared to that in the age group 15–24 years where it was 10.8% (*x* ^2^ test, p<0.01). Respondents of Muslim background had HIV infection prevalence of 14.4% compared to 25.2% among Christians (*x* ^2^ test, p<0.01). HIV infection was significantly lower among the unmarried respondents (14.4%) than in the married people (25.2%), *x* ^2^ test, p<0.01, and highest amongst the widowed respondents. The uncircumcised men had HIV prevalence of 27.2% compared to 10.6% in the circumcised men (*x* ^2^ test p<0.01). There was no statistically significant bivariate association between HIV infection and sex, education, distance from former place of residence, mobility during the last 12 months preceding the survey and comprehensive knowledge of HIV.

**Table 3:** Estimated prevalence of HIV by HIV-related variables among fishing community members age 15–49 years.

Variable	HIV prevalence (%)	95% Confidence Interval	Significance p-value	Number of respondents
**Sex**			**0.086**	
Female	25.5	19.4–31.5		326
Male	20.4	15.6–25.2		510
**Age**			0*	
15–24 years	10.8	5.4–16.3		203
25 years and above	26.1	21.4–30.7		633
**Religion**			0.003*	
Muslim	14.4	8.7–20.0		167
Christian	25.2	20.4–30.0		618
**Education**			0.091	
Primary or below	23.1	18.3–27.9		537
Above Primary	17.5	11.3–23.8		217
**Marital Status**			0*	
Never married	6.2	1.0–11.4		81
Ever married	24.1	19.9–28.4		755
**Distance to Former Place of Residence**			0.6	
Less than 100 kilometers	23.8	19.0–28.6		424
100 kilometers and above	22.1	15.8–28.5		298
**Mobility during last 12 months; whether ever slept away**			0.644	
Ever slept away	22.9	18.2–27.5		547
Never slept away	21.5	16.0–26.9		289
**Duration of staying away from home during last 12 months**			0.625	
Ever been away for a month	21.0	13.0–28.9		167
Never been away for a month	22.7	18.4–27.0		669
**Comprehensive Knowledge Status**			0.733	
Have comprehensive knowledge	23.0	17.0–29.0		313
Have no comprehensive knowledge	22.0	17.8–26.2		523
**Circumcision Status**			0*	
Circumcised	10.6	7.2–13.9		208
Uncircumcised	27.2	20.8–33.6		294

**Footnote:** Chi-square (χ^2^) statistical test of significance was used. The results of significant p-values were corroborated using odds ratio.

We also analyzed the relationship between HIV infection and HIV-related behavior variables and reported STI. Table 4 shows bivariate association with HIV sero-status among men and women age 15–49 years. The findings show that ever use of condoms and number of lifetime sexual partners had significant association with HIV infection. Unexpectedly, respondents who reported ever using condoms had HIV infection prevalence of 25.1% compared to 16.5% among those who reported not using condoms (*x* ^2^ test p<0.05). HIV prevalence was higher among respondents who reported 3 or more lifetime sexual partners compared to 10.7% among respondents reporting 1 to 2 lifetime sexual partners (*x* ^2^ test p<0.01). There was no statistically significant association between HIV infection and age at first sex, number of sexual partners in the last one month, higher risk sex, condom use in the last 12 months, condom use at last higher risk sex and reported STI.

**Table 4:** Bivariate association with HIV sero-status among men and women age 15–49 years.

Variable	HIV prevalence (%)	95% Confidence Interval	Significance p-value	Number of respondents
**Age at first sex**			0.684	
Less than 18 years	23.3	18.4–28.2		524
18 years and above	22.0	16.9–27.1		286
**Ever use of condom**			0.011*	
Ever used condom	25.1	20.3–29.9		598
Never used a condom	16.5	11.0–22.0		212
**Number of lifetime sexual partners**			0*	
1–2 partners	10.7	4.9–16.4		159
3+ partners	25.4	21.0–29.8		637
**Number of sexual partners in the last 12 months**			0.994	
Less than 2 partners	22.8	18.2–27.5		569
2+ partners	22.8	16.8–28.8		241
**Higher risk sex in last 12 months**			0.266	
Had higher risk sex	22.8	18.2–27.5		266
Had no higher risk sex	22.8	16.8–28.8		544
**Condom use at last sex in 12 months**			0.109	
Used condom at last sex	27.1	20.0–34.1		192
Did not use condom at last sex	21.5	16.9–26.1		618
**Condom use in higher risk sex in past 12 months**			0.716	
Used condom in high risk sex	24.3	16.7–32.0		148
Did not use condom in high risk sex	26.3	17.9–34.7		118
**Women reporting an STI**			0.108	
Reported having STI	19.2	10.1–28.4		78
Did not report having STI	28.5	21.5–35.4		239
**Men reporting an STI**			0.315	
Reported having STI	24.5	14.9–34.1		94
Did not report having STI	19.8	13.9–25.7		399

**Footnote:** Chi-square (χ^2^) statistical test of significance was used. The results of significant p-values were corroborated using odds ratio.

#### Multivariate analysis

Table 5 summarizes the results of a multivariate analysis done to identify risk factors for HIV infection in fishing communities. Three models were constructed, namely; one for both women and men, one for men only and one for women only.

**Table 5:** Risk factors for HIV infection in fishing communities (Multivariate analysis).

Variable	Unadjusted Odds Ratio		Adjusted Odds Ratio	
Both men and women				
	Value	95% C.I	Value	95% C.I
**Age**				
15–24 years	Ref	Ref	Ref	Ref
25 years and above	2.9	1.8–4.7	2.4	1.4–4.0
**Religion**				
Moslem	Ref	Ref	Ref	Ref
Christian	2.0	1.3–3.2	2.0	1.2–3.3
**Ever use of condom**				
Ever used condom	Ref	Ref	Ref	Ref
Never used a condom	0.6	0.4–0.9	0.6	0.3–0.9
**Number of lifetime sexual partners**				
1–2 partners	Ref	Ref	Ref	Ref
3+ partners	2.8	1.7–4.9	2.3	1.3–4.1

Men only				
**Age**				
15–24 years	Ref	Ref	Ref	Ref
25 years and above	5.1	2.3–11.3	3.5	1.4–8.6
**Circumcision Status**				
Circumcised	Ref	Ref	Ref	Ref
Uncircumcised	3.2	1.9–5.3	3.1	1.8–5.4

Women only				
**Ever use of condom**				
Ever used condom	Ref	Ref	Ref	Ref
Never used a condom	0.3	0.2–0.6	0.4	0.2–0.8
**Number of lifetime sexual partners**				
1–2 partners	Ref	Ref	Ref	Ref
3+ partners	3.3	1.7–6.3	3.4	1.7–6.9

##### Model 1 Both women and men

Age, religion, ever use of condoms and number of lifetime sexual partners were found to be predictors for HIV infection. Respondents who were 25 years or older were 2.4 times more likely to have HIV infection compared to those aged 15–24 years (95% CI Adjusted OR 1.4–4.0). Respondents of Christian background were 2 times more likely to have HIV infection compared to Muslims (95% CI Adjusted OR 1.2–3.3). An unexpected effects was seen in ever use of condom, where it was shown not to be protective; those who reported ever use of condoms were about 1.7 times more likely to have HIV compared to those that have never used a condom in their lifetime (95% CI Adjusted OR 0.3–0.9). Number of lifetime sexual partners was a predictor (95% CI Adjusted OR 1.3–4.1), those who reported to have had 3 or more partners in their life time were 2 times more likely to have HIV compared to those that limited lifetime partners to not more than two.

##### Model 2 Men only

Two factors were found to be associated with HIV infection; they include age and circumcision status. Males age 25 years and above were 3.5 times more likely to have HIV compared to those age 15–24 years (95% CI Adjusted OR 1.4–8.6). Circumcision was protective; men who were not circumcised were 3.1 times more likely to be infected with HIV compared to those who were circumcised (95% CI Adjusted OR 1.8–5.4).

##### Model 3 Women only

After controlling for confounders, two variables were found to be associated with HIV infection. Respondents who reported to have had 3 or more sexual partners in their lifetime were 3.4 times more likely to have HIV infection compared to those that limited lifetime partners to not more than two (95% CI Adjusted OR 1.7–6.9). Paradoxically, women who had ever used a condom were about 2.5 times more likely to have HIV compared to those that have never used a condom in their lifetime (95% CI Adjusted OR 0.2–0.8).

## Discussion and Conclusions

This study is one of the few studies that have shed light on the magnitude and risk factors of HIV infection among fishing communities in Uganda. The HIV prevalence estimate of 22% is three times higher than the estimated HIV prevalence of 7.3% for the general adult population in Uganda [5]; thus showing that fishing communities are at a substantially higher risk of acquisition/transmission of HIV infection.

In pursuing another objective of this study, we earlier documented that fishing communities in the areas surveyed are underserved with HIV services compared to other communities [10]. Specifically, we indentified glaring gaps in services for PMTCT, HCT, STD Management, SMC and ART. To address these gaps, we underscored the need to scale-up and repackage these services. We also recommended that efforts should be made to bridge the gap between the high burden of HIV infection in fishing communities and the unavailability/inadequacy of HIV prevention, care and support services. If the gaps are not addressed, fish landing sites will continue to pose a very big challenge to the reduction of HIV transmission in the country. The gaps could be filled through innovative implementation approaches that enhance collaboration and partnership to deliver HIV combination prevention interventions. Services could be extended to remote and hard to reach communities using outreach or mobile HIV service outlets.

The HIV prevalence of 22 percent found in this survey reaffirms the categorization of fishing communities as one of the most-at-risk-populations (MARPs). We previously reported higher HIV prevalence among MARPs in Uganda, including men who have sex with men (MSM), commercial sex workers (CSWs) and partners of CSWs [11]. These communities live in unique environments with limited social amenities; due to this, they have special social needs.

The attitudes of fishing communities may also affect the uptake of HIV prevention services. For instance, fishing communities have been reported to have fatalistic attitudes; with some of them viewing HIV infection as less risky than drowning during fishing [7]. Because of this, their concern do center on survival in water than to taking preventive measures against HIV infection. We recommend that this type of attitude of refusal to change behaviors despite being aware of the potential fatal consequences of HIV infection should be tackled by promoting positive attitudes about life. Furthermore, due to some uniqueness of the attitudes, behaviors and other characteristics of fishing communities, we recommend that tailored made HIV prevention, care and support services be designed to target fishing communities.

This study demonstrated that uncircumcised men were at increased risk of HIV transmission. Therefore, there is need to scale up medical male circumcision services in fishing communities. In Uganda, safe male circumcision has been adopted as a component of the HIV combination prevention package and its delivery is now a government policy [12], [13]. The survey also showed that multiple sex partnership is a risk factor for HIV infection among the surveyed communities. In view of this, we recommend that programmes that promote HIV risk avoidance or risk reduction be established where they do not exist and strengthened where they are weak.

This study had one limitation. Forty six fish landing sites around the Lake Victoria Basin of Uganda were surveyed. Fish landing sites from other regions such as those in the Lake Albert, Lake Kwania and Lake Kyoga basins were not considered. Therefore, although the results obtained from the survey are representative of fishing communities of the Lake Victoria Basin of Uganda, they are not representative of all fishing communities in Uganda. Never-the-less, a number of general findings and the recommendations made are likely to be relevant to any fishing community in the whole of Uganda and the region.
